# Synchronous or Metachronous Hairy Cell Leukemia and Chronic Lymphocytic Leukemia: A Case Series and Literature Review

**DOI:** 10.3389/fonc.2016.00270

**Published:** 2017-01-09

**Authors:** Vivek Verma, Smith Giri, Vijaya Raj Bhatt, Catalina Amador-Ortiz, James O. Armitage

**Affiliations:** ^1^Department of Radiation Oncology, University of Nebraska Medical Center, Omaha, NE, USA; ^2^Department of Medicine, University of Tennessee Health Science Center, Memphis, TN, USA; ^3^Division of Internal Medicine, Department of Hematology and Oncology, University of Nebraska Medical Center, Omaha, NE, USA; ^4^Department of Pathology and Microbiology, University of Nebraska Medical Center, Omaha, NE, USA

**Keywords:** hairy cell leukemia, chronic lymphocytic leukemia, cladribine, rituximab, fludarabine

## Abstract

**Introduction:**

Concurrent hairy cell leukemia (HCL) and chronic lymphocytic leukemia (CLL) is rare; management is inadequately described in the literature.

**Methods:**

Retrospective chart review and clinical follow-up.

**Results:**

Five patients are described. The first patient developed synchronous HCL and CLL and was treated with rituximab for 13 months with HCL in remission and stable CLL. The second patient developed HCL and was treated with cladribine. His disease recurred 7 years later which was retreated with cladribine. Seven years later, he developed asymptomatic CLL. The third patient developed CLL, managed expectantly, then developed HCL 10 months later, and was treated with cladribine. Although his HCL went into remission, there was a slow redevelopment of CLL for which expectant management was done. The fourth patient developed concurrent CLL and HCL, received cladribine, with subsequent development of worsening abdominal lymphadenopathy and was lost to follow-up. The last patient developed concurrent HCL and CLL and was also diagnosed with lung adenocarcinoma; this patient was also lost to follow-up.

**Conclusion:**

The development of concurrent HCL and CLL may indicate a common origin. Patients with HCL may subsequently develop CLL, thus mimicking a relapse of HCL. Therapy requires individualized approach including watchful waiting in asymptomatic cases. Rituximab may be useful to treat both disorders simultaneously.

## Introduction

This work was approved by the University of Nebraska Medical Center Institutional Review Board and Ethics Committee. Ethical approval was obtained for the study and all participants have consented to participate in the research project and to the publication of their personal communication. All research participants signed written informed consent in accordance with the Declaration of Helsinki.

### Case 1

A 69-year-old Caucasian male, with a history of 35 pound weight loss, was found to have a white blood cell (WBC) count of 20.8 × 10^9^/L, with 79% lymphocytes. His brother had died of chronic lymphocytic leukemia (CLL). Flow cytometric analysis revealed two distinct monoclonal B-cell populations. A predominant CD19+/CD20−/CD5+/CD23+/dim surface kappa+/CD25−/CD103− population, consistent with CLL and the second (bright CD20+/bright CD19+/CD11c+/CD25+/CD103+/bright lambda+) smaller population, consistent with hairy cell leukemia (HCL). Bone marrow (BM) biopsy was predominantly involved by HCL, with focal involvement by CLL. Cytogenetic analysis showed a deletion of 7q31. He underwent four treatments of weekly rituximab (375 mg/m^2^) followed by maintenance rituximab every 3 months for a total of 13 months. Two months after completion of treatment, flow cytometry of the peripheral blood showed involvement by the CLL cell population, but no evidence of HCL. He was then clinically monitored for CLL owing to a lack of symptoms and stable WBC counts between 10 and 16 × 10^9^/L. The patient developed papillary thyroid cancer 51 months after initial diagnosis for which he had successful surgery. Fifty-eight months after initial diagnosis, CT chest was found to have multiple pulmonary and pleural nodules, and while further workup was underway, he was hospitalized with dyspnea and altered mental status and passed away (Table 1).

**Table 1:** **Clinical characteristics of the study population**.

Case	Age at first diagnosis	First diagnosis	CBC	LDH (U/L) (normal range)	Cytogenetics	Treatment	Outcome	Follow-up (months)
1	69	HCL, CLL	WBC 20.8 (Ly 79%)	100 (98–192)	Deletion of 7q31	Rituximab	HCL in remission; stable CLL	58
			Hgb 15.8					
			Plt 108					

2	49	HCL^a^	WBC 11.1 (Ly 71%)	563 (313–618)	Trisomy 12	Cladribine	HCL relapse; development of CLL	250
			Hgb 13.9					
			Plt 113					

3	47	CLL^b^	WBC 3.1	–	Deletion of 13q	Cladribine	HCL in remission; slow redevelopment of CLL with expectant management thereafter	79
			Hgb 12.3					
			Plt 52					

4	43	HCL, CLL	WBC 4.1 (Ly 34%)	130 (98–192)	Trisomy 12, partial deletion of 5′ telomeric 14q32 locus	Cladribine	Worsening lymphadenopathy; lost to follow-up	13
			Hgb 14.1					
			Plt 94					

5	79	HCL, CLL	WBC 9.0 (Ly 68%)	–	None	None^c^	Transfer to an outside facility	1
			Hgb 18.7					
			Plt 102					

*CBC, complete blood count; HCL, hairy cell leukemia; CLL, chronic lymphocytic leukemia; WBCs, white blood cells (thousands of cells per microliter); Ly, lymphocytes; Hgb, hemoglobin (grams per deciliter); Hct, hematocrit; Plt, platelets (thousands of cells per microliter); SLL, small lymphocytic lymphoma*.

*^a^In this patient, HCL relapsed after 7 years and was retreated with cladribine. The patient developed CLL 14 years after the initial diagnosis of HCL*.

*^b^In this patient, CLL was diagnosed with HCL 10 months after initial diagnosis of CLL*.

*^c^The patient was diagnosed with lung cancer and underwent therapy for lung cancer*.

### Case 2

A 49-year-old Caucasian female developed HCL treated with cladribine which led to remission. She relapsed 7 years later and was successfully retreated with cladribine. Seven years thereafter, she presented with cervical lymphadenopathy. WBC count was 11.1 × 10^9^/L with 71% lymphocytes. Biopsy of the lymph node revealed small lymphocytic leukemia (SLL) and a subsequent BM biopsy showed involvement by CLL/SLL occupying 10% of the BM space. At that time, HCL was not detected in the peripheral blood or BM biopsy. Cytogenetic analysis showed trisomy 12. She is being clinically followed up in our clinic and remains asymptomatic and clinically stable at 82 months after CLL diagnosis (nearly 21 years after initial HCL diagnosis).

### Case 3

A 49-year-old Caucasian male was referred for pancytopenia and spontaneous bruising. Complete blood count (CBC) showed a WBC count of 3.1 × 10^9^/L and platelets of 52 × 10^9^/L. A peripheral blood smear and BM biopsy performed at an outside institution were reportedly normal, but flow cytometry revealed a dim CD19+/CD20+/CD5+/CD23+/kappa+ monoclonal B-cell population, consistent with a monoclonal B-cell lymphocytosis of CLL phenotype. Cytogenetic analysis revealed 13q deletion. Clinical expectant management and close follow-up was instituted due to patient preference. Ten months later, he presented with febrile neutropenia. A peripheral blood smear at that time showed circulating lymphoma cells, morphologically consistent with simultaneous involvement by HCL and CLL. A subset of the neoplastic cells showed tartrate resistant acid phosphatase (TRAP) positivity. A BM biopsy showed diffuse involvement by HCL, and no evidence of involvement by CLL. The patient was treated with cladribine for a month with a repeat BM biopsy 2 months after completion showing resolution, and confirmed by normalization of blood counts and flow cytometry. He was clinically followed up for the next 5.5 years, until he started developing thrombocytopenia (hemoglobin 13.0 g/dL, platelets 104 × 10^9^/L, and WBC 7.8 × 10^9^/L). A BM biopsy showed simultaneous involvement by HCL and CLL (20 and 5% of the BM space, respectively). A 13q deletion was present. The patient was managed expectantly.

### Case 4

**Figure 1:**
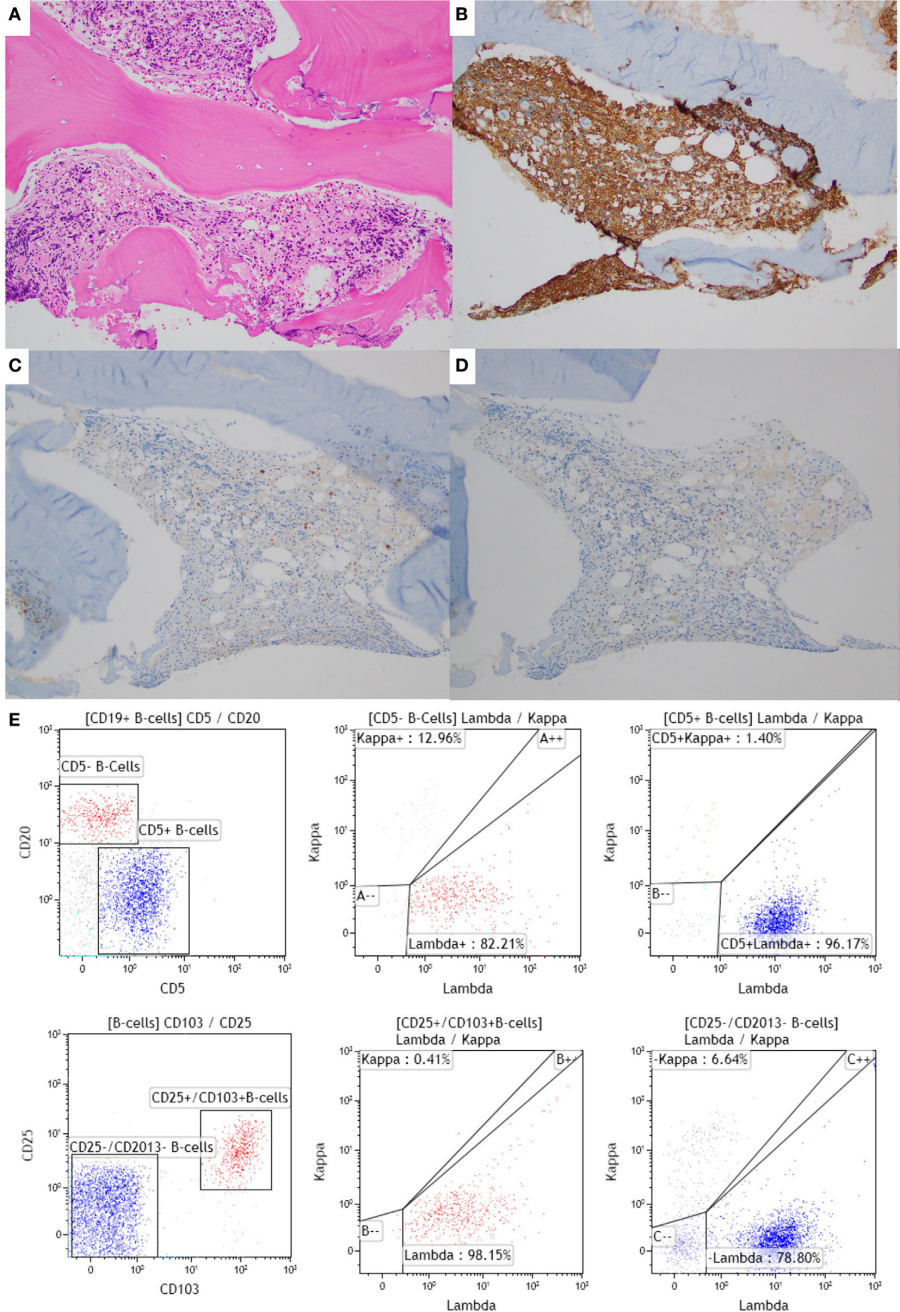
**Bone marrow (BM) biopsy of a case with synchronous chronic lymphocytic leukemia (CLL)/small lymphocytic leukemia (SLL) and hairy cell leukemia (HCL)**. **(A–D)** BM core biopsy specimen showing an abnormal lymphoid infiltrate with a diffuse growth pattern. The infiltrate is positive for CD20 **(B)**, and negative for CD5 and CD23 [**(C,D)**, respectively], consistent with involvement by HCL. The CLL infiltrate is focally present in the core biopsy (1% of the BM space; data not shown). **(E)** The corresponding flow cytometric analysis shows two distinct monoclonal populations: a CD20 dim+, CD5+, CD103−, CD11c−, lambda restricted CLL/SLL population (in blue) and a CD20 bright+, CD5−, CD103+, CD11c+, lambda restricted HCL population (in red). The HCL population is also positive for CD25 (data not shown).

A 43-year-old male with a family history of leukemia in his grandmother and multiple myeloma in his grandfather experienced night sweats and left upper quadrant abdominal pain, and presented to an outside facility. A CBC showed monocytopenia, and peripheral smear showed rare atypical lymphoid cells, suggestive of CLL. Flow cytometry of the peripheral blood showed a predominant population of mature B-cells (CD19+/CD20+/CD5+/CD23+/moderate-density lambda+) and the second very small population (bright CD19+/bright CD20+/bright CD11c+/CD25+/CD103+/moderate lambda+), consistent with HCL. BM biopsy then demonstrated HCL, involving 20% of the BM space and CLL, involving 1% of the BM space (Figure 1). Flow cytometry of the BM aspirate detected the two distinct monoclonal B-cell populations with similar immunophenotypic characteristics to that seen in the peripheral blood. Cytogenetic studies showed trisomy 12 and a partial deletion of the 5′ telomeric 14q32 locus. He was treated with 7 days of intravenous cladribine, resulting in remission of HCL. Just over a year later, he experienced abdominal swelling and right upper quadrant abdominal pain, with computed tomography revealing retroperitoneal and mesenteric lymph nodes that were pathologically demonstrated to be HCL. The patient was subsequently lost to follow-up.

### Case 5

A 79-year-old Caucasian male presented with 1 year history of dry cough and a 15-pound weight loss. Chest imaging revealed multiple lung nodules, which were biopsied and showed adenocarcinoma. CBC showed WBC of 9.0 × 10^9^/L with 68% lymphocytes and hemoglobin of 18.0 g/dL. Peripheral smear showed two distinct neoplastic populations. The majority of lymphocytes showed cytological features of CLL and were admixed with a few hairy cells. The BM aspirate showed a reverse pattern with the majority of cells having cytological and immunophenotypic features of HCL (diffuse interstitial infiltrate of strong CD20+/CD5−/DBA-44+ B-cells). In addition, flow cytometry of the BM aspirate showed two distinct monoclonal B-cell populations, a CD19+/dim CD20+/dim CD5+/CD23+/dim kappa+ population, consistent with CLL and a bright CD19+/bright CD20+/CD11c+/CD25+/CD103+/bright kappa+ population, consistent with HCL. No cytogenetic abnormalities were detected. Given findings of multiple pulmonary nodules and borderline enlarged mediastinal lymph nodes, it was recommended that the patient undergo chemotherapy for lung cancer first. However, the patient was transferred to an outside facility.

## Background

In this paper, we describe five patients with coexistence of HCL and CLL seen at our institution and focus on differential pathologic, immunophenotypic, and cytogenetic characteristics, as well as examine their treatment regimens and outcomes.

Chronic lymphocytic leukemia and HCL are chronic B-cell lymphoproliferative disorders with distinct morphologic features, immunophenotype, and clinical presentations. The diagnosis of CLL is usually based on the finding of lymphocytosis and the presence of a monotonous population of small lymphocytes with clumped chromatin and scant cytoplasm in the peripheral blood smear. These tumor cells express CD5, CD23, dim monotypic surface immunoglobulin, and dim CD20. Lymphadenopathy is frequent, and splenomegaly is seen in a relative minority of patients with CLL (1). Conversely, HCL presents with pancytopenia (or monocytopenia), splenomegaly, and opportunistic infections. Lymphadenopathy is unusual in HCL. Most patients do not present with lymphocytosis, but circulating intermediate-sized neoplastic lymphoid cells with characteristic “hairy” cytoplasmic projections are often present (2). The classic immunophenotypic profile of HCL consists of bright expression of CD19, CD20, CD22, and monotypic surface immunoglobulin and expression of CD103, CD25, CD11c, AnnexinA1, DBA.44, and cyclin D1 (usually weak). Additionally, all HCL cases will contain at least some cells with cytoplasmic TRAP positivity (1–6).

The association of HCL and CLL is considered to be rare (7), with prior experiences limited to a few case reports (7–10). It is increasingly being acknowledged that HCL can predispose to developing multiple types of secondary malignancies including non-Hodgkin lymphomas, possibly due to associated cancer susceptibility and/or treatment-related effects (11–13), although the etiology is debated (14). Although a high frequency of non-Hodgkin lymphomas have been reported (12, 14), the occurrence of CLL as the second malignancy has only been reported by one study (12).

The presence of concurrent or metachronous CLL and HCL can predictably be problematic for clinicians given its rarity. Treatment can be a challenge, given relatively indolent courses for both malignancies and balancing it against the potential complications of therapy. Such treatment decisions are often left for a case-by-case basis, due to the need to gauge the overall clinical circumstance in deciding treatment and follow-up options.

## Discussion

Concurrent diagnosis of HCL and CLL has been rarely described in the literature; as a result, the characteristics, treatment options, and prognosis remain unclear. We characterize five patients seen at our institution that developed HCL and CLL including pathologic findings, management, and outcomes.

Our results are in many aspects both similar to and different from prior case reports. Gine et al. described three patients with concurrent HCL and CLL, all of whom had two distinct monoclonal CD5+/CD23+ and CD11c+/CD25+ (and two with CD103+) B-cell populations. Two patients presented with features of HCL (splenomegaly and pancytopenia) and the diagnosis of CLL was made based on morphologic and immunophenotypic assessment of the monoclonal B lymphocytes. The third patient was found to have CLL, 1 year following the diagnosis of HCL. Cytogenetics was normal in two patients, whereas one patient had a t(1;19)(p35;q40) translocation. All three patients were initially treated with pentostatin, which led to remission of HCL. However, two out of three patients subsequently exhibited progression of CLL and were treated successfully with cladribine and fludarabine/cyclophosphamide/mitoxantrone followed by autologous stem cell transplant, respectively (7).

Brown et al. reported a 75-year-old man who developed metachronous HCL 17 years following diagnosis and treatment of CLL. The patient was initially treated with chlorambucil and prednisolone due to worsening night sweats, splenomegaly, and pancytopenia. However, pancytopenia persisted, and he underwent splenectomy. He was found to have both CLL and HCL in his BM 18 years following his initial diagnosis. Flow cytometric analysis revealed two distinct clonal lymphocytes; a low-frequency CD19+/CD5+/FMC7−/CD25−/dim surface immunoglobulin+ B-cell population consistent with CLL and a high-frequency FMC7+/CD25+/CD22+/CD5+/bright surface immunoglobulin+ B-cell population consistent with HCL. Interestingly, the HCL clone was also CD5+, a finding that has been reported previously (15). Subsequently, the patient was treated with alpha interferon, which resulted in remission and no evidence of recurrence at 19 months of follow-up (8).

Finally, Sokol and Agosti demonstrated two different monoclonal populations in the peripheral blood of an 83-year-old male, a dominant CD19+/CD20+/CD5+/CD23+ clone with lambda light chains, and a minor CD20+/CD11c+/CD25+/CD103+ clone with kappa restriction, consistent with concurrent CLL and HCL. Chlorambucil and prednisone yielded no response, and subsequent cladribine (one cycle) led to stable disease for 6 months. Three cycles of fludarabine and cyclophosphamide yielded complete response for 5–7 months before severe anemia led to the discovery of CLL infiltration of the BM. He was then treated with eight weekly cycles of rituximab, which led to a complete response at 8 months follow-up (10).

The precise reason for coexistence of both CLL and HCL is unknown. Both HCL and CLL are chronic B cell lymphoproliferative disorders that represent malignant transformations of B lymphocytes at different stages of a single differentiated pathway (8). Gene expression profiling studies have suggested that HCL patients may originate from post-germinal center memory B cells (16). Conversely, about 50% of CLL derive from post-germinal center CD5+, CD27+, and memory B cells (7, 17). Transformations of CLL to B cell lymphoproliferative disorders such as large cell lymphoma and prolymphocytic leukemia are a well-recognized phenomenon. Similarly, there is also evidence that HCL can also evolve into higher grade lymphoma, much like the Richter’s transformation seen among patients with CLL (18). Patients with CLL have impaired immune surveillance that makes them prone to develop second neoplasms. Similarly, patients with HCL have been found to have an excess risk of developing hematologic malignancies, particularly Hodgkin and non-Hodgkin lymphomas (12). Presence of the same immunoglobulin gene rearrangements in two different clonal population (HCL and CLL) may suggest a common precursor origin and has been reported before (7, 9). However, other reported cases presented with two different immunoglobulin rearrangements, suggesting a different clonal origin (7, 8, 10). Moreover, emerging data from a recent study emphasize sequencing of the IGHV-D-J and BRAF V600E loci (19). It is important to note that leukemic cells from CLL patients, upon exposure to phorbol ester, acquire the phenotype of hairy cells (20). In addition, chronic B cell lymphoproliferative disorders with features intermediate between HCL and CLL have described in the literature (21, 22). These observations suggest that HCL and CLL may be clonal disorders at different stage of maturation of the same lymphocyte.

Our case series includes patients with both synchronous (patients 2, 3, 4, and 5) and metachronous (patient 1) existence of HCL and CLL. Most of the prior reports have included patients with coexistence of HCL and CLL (7, 10), although both HCL developing among CLL patients (8) and CLL developing among HCL patients (7), have been described. Hisada et al. had earlier reported 5 cases of secondary CLL among 3,104 patients with HCL in the Surveillance, Epidemiology, and End Results Program database (12). Similarly, population-based studies have demonstrated an increased risk of secondary hematological malignancies, particularly lymphomas, among patients with a history of CLL. However, an increased risk of HCL has not been specifically evaluated in these studies (23, 24).

A common theme among patients with synchronous tumors is that peripheral blood flow cytometry often reveals CLL to be the dominant clone, whereas HCL is often dominant on BM studies. Brown et al. suggest that the dominance of HCL in BM may be reflective of a growth advantage of HCL over CLL. Furthermore, the authors hypothesize that this could be due to differential rates of proliferation or apoptosis, a secondary immunodeficiency state allowing emergence and progression of the second lymphoproliferative disorders and inhibitory cytokine production by HCL cells (8). In light of these observations, it is interesting to note that in our series, most patients required treatment for HCL, whereas CLL was often asymptomatic and slowly progressive and could be followed up alone.

The diagnosis of simultaneous CLL and HCL based on morphology alone can be challenging. In our case series, HCL was the predominant component in the BM. Flow cytometry and immunohistochemical stains were required to highlight the more subtle CLL component. The opposite can also be the case since it is well known that HCL infiltrates can be subtle, especially when they show an interstitial growth pattern. Given the distinctive immunophenotype of HCL and CLL, flow cytometry and immunohistochemistry are invaluable tools in the simultaneous diagnosis of these entities. However, since it is well known that HCL is associated with increased reticulin fibrosis and often results in a “dry tap”; flow cytometry of the BM may not always be available (25). In these cases, concurrent flow cytometry of the peripheral blood can be helpful in identifying the distinct populations. Additionally, a lymph node biopsy can confirm the diagnosis of CLL.

Though our approach to treatment in these patients centered around cladribine and/or rituximab, other reports have demonstrated acceptable results with pentostatin (7), alpha-interferon (8), and fludarabine/cyclophosphamide followed by rituximab (10). In light of previous case reports demonstrating failure sometimes after multiple chemotherapy regimens, it is noteworthy in our analysis that simple expectant management of select patients resulted in long-term survival without clinical consequence.

## Conclusion

We report our institutional experience on five patients with coexistence of HCL and CLL, along with a review of previously reported cases. Concurrence of HCL and CLL may suggest an underlying relationship, and these two malignancies possibly represent clonal disorders at different stage of maturation of the same lymphocyte. Our experience suggests a variable presentation, chronology, and a need to tailor treatment strategy based on relative activity among these two diseases.

## Author Contributions

VV and SG performed data analysis, CA-O performed pathological analysis and image acquisition, and VB and JA played supervisory roles. All the authors wrote, read, and approved the manuscript.

## Conflict of Interest Statement

JA reports receiving consulting fees from Ziopharm Oncology, GlaxoSmithKline IDMC, Spectrum Pharmaceuticals, Roche, Conatus—IDMC, and serving on the board of directors for Tesaro bio Inc. There are no conflicts of interest for any other authors.
